# *Candida* species distribution, genotyping and virulence factors of *Candida albicans* isolated from the oral cavity of kidney transplant recipients of two geographic regions of Brazil

**DOI:** 10.1186/1472-6831-14-20

**Published:** 2014-03-15

**Authors:** Walicyranison Plinio da Silva-Rocha, Vitor Luiz de Brito Lemos, Terezinha Inês Estivalet Svidizisnki, Eveline Pipolo Milan, Guilherme Maranhão Chaves

**Affiliations:** 1 Laboratório de Micologia Médica e Molecular, Departamento de Análises Clínicas e Toxicológicas, Universidade Federal do Rio Grande do Norte, Natal, Rio Grande do Norte, Brazil; 2 Departamento de Infectologia, Universidade Federal do Rio Grande do Norte, Natal, Rio Grande do Norte, Brazil; 3 Departamento de Análises Clínicas, Universidade Estadual de Maringá, Maringá, Paraná, Brazil; 4 Universidade Federal do Rio Grande do Norte, Centro de Ciências da Saúde, Departamento de Análises Clínicas e Toxicológicas, Laboratório de Micologia Médica e Molecular. Rua Gal. Gustavo Cordeiro de Faria S/N. Petrópolis, Natal, Rio Grande do Norte, Brazil

**Keywords:** *Candida* spp, Oral candidiasis, Kidney transplant recipients, Genotyping, Virulence factors

## Abstract

**Background:**

*Candida albicans* is a diploid yeast that in some circumstances may cause oral or oropharyngeal infections. This investigation aimed to study the prevalence of *Candida* spp. and to analyze the ABC genotypes of 76 clinical isolates of *C. albicans* obtained from the oral cavity of kidney transplant patients from two distinct geographic regions of Brazil.

**Methods:**

We typed 48 strains with ABC genotyping and Microsatelitte using primer M13 and tested three virulence factors in vitro: phospholipase activity, morphogenesis and the ability to evade from polymorphonuclear neutrophils phagocytosis.

**Results:**

*C. albicans* was the most prevalent species (86.4%), followed by *C. tropicalis* (4.5%). *C. albicans* genotype A was the most prevalent (58 isolates; 76.4%), followed by genotype C (15 isolates; 19.7%) and genotype B (3 isolates; 3.9%). When Microsatellite technique with primer M13 was applied, 80% of the isolates from the South were placed within the same cluster. The majority of Genotype C strains were grouped together within two different clusters. Genotype C was considered more resistant to PMNs attack than genotypes A and B. Strains isolated from the South of Brazil showed also better ability to combat PMNs phagocytosis.

**Conclusions:**

We found a high rate of *C. albicans* genotype C strains isolated from the oral cavity of this group of patients. This study characterized oral *C. albicans* strains isolated from kidney transplant recipients and will contribute to a better understanding of the pathogenesis of oral candidiasis.

## Background

Despite the fact that *Candida* spp. belong to the normal oral microbiota, living on the tongue, gums, palate and saliva of healthy individuals as commensal yeasts, they can cause oral or oropharyngeal infections [1,2]. *Candida* spp. have been isolated from 40 to 60% of healthy mouths, whereas oral candidiasis is more common in immunodeficient individuals, those with severe underlying diseases, and upper denture wearers [3,4].

Despite the fact that other less virulent *Candida* species such as *C. glabrata, C. tropicalis, C. parapsilosis, C. krusei* and *C. dubliniensis* have also been isolated from the saliva of patients with or without oral candidiasis, *C. albicans* is still the most frequent species associated with oral lesions [5].

Several factors may contribute for the transition from commensal yeasts to pathogenic forms which are related to impaired host immune system associated with the virulence of the micro-organism. The main virulence factors of *C. albicans* include bud-to-hypha transition (also called morphogenesis), adhesion to human epithelial and endothelial cells, biofilm formation, the ability to secrete hydrolytic enzymes (mainly proteinases and phospholipases), phenotypic switching (white-opaque transition) as well as evasion of host immune cells [6,7].

Some studies have evaluated *C. albicans* ABC genotypes isolated from the oral cavity of different individuals and the results seem to be very variable, according to the population evaluated. For instance, a study with 11 diabetic patients with periodontitis revealed that 51.6% subgingival *C. albicans* isolates belonged to genotype B [8], while other investigation has found genotype A as predominant [9].

The prevalence of oral candidiasis in renal transplant recipients described in the literature ranges from 9.4% to 46.7% [10-12]. A study performed in Mexico by De La Rosa-Garcia et al [11], reports 18.7% of cases of oral candidiasis in a total of 90 kidney transplanted recipients.

Our group has previously published a comparison of some virulence factors (biofilm production, adhesion to human buccal epithelial cells and proteinase activity) among *C. albicans* and non-*C. albicans Candida* isolates obtained from the oral cavity of kidney transplant recipients from Natal, Rio Grande do Norte, Brazil [13]. This study is a follow up of this previous publication including other strains belonging to another geographic region of Brazil. In addition, other virulence attributes were investigated. Therefore, the objectives of the present study were to determine *Candida* species distribution and to genotypically characterize 48 *C. albicans* strains isolated from the buccal cavity of 154 kidney transplanted recipients from two different geographic regions of Brazil (Northeast and South). In addition, the strains were phenotypically characterized for the ability to express the following virulence factors in vitro: The secretion of phospholipase, the transition from yeast cells to hyphae (morphogenesis) and the ability to resist to phagocytosis by polymorphonuclear neutrophils.

## Methods

### Strains selection

During a 3 months-period, 154 patients from two different geographic regions of Brazil (111 from Natal, Rio Grande do Norte and 43 from Maringa, Parana) were subjected to clinical examination of the oral cavity for candidiasis diagnosis, according to the criteria established for oral lesions in HIV patients recommended by the EC-Clearinghouse and World Health Organization classifications [14]. Only patients who agreed to take part on a surveillance confidential study, in accordance to the Local Research Ethics committee from The Onofre Lopes University Hospital, approved under the number 152/07, were enrolled in this study. Strains isolates from oral colonization (without any symptoms) or during episodes of oral candidiasis were evaluated in this study. For genotyping and virulence studies, we randomly selected 48 clinical strains of *C. albicans* (38 from Natal, Rio Grande do Norte and 10 from Maringa, Parana). All the isolates are described in Table 1. The samples were stocked on YPD glycerol (10 g/L yeast extract, 20 g/L peptone, 20 g/L dextrose and 3% glycerol) at -80°C at the Laboratório de Micologia Médica e Molecular, Universidade Federal do Rio Grande do Norte, Brazil. *C. albicans* ATCC90028 and SC5314 were included in this study as reference strains.

**Table 1:** Demographic data of kidney transplant recipients from Natal, RN and Maringa, PR, Brazil, according to gender, age, and underlying disease

**Variables**	**Natal (n,%)**	**Maringa (n,%)**	**Total (n,%)**
*Sex*			
Male	64 (73.6)	23 (26.4)	87 (100)
Female	47 (70.1)	20 (29.9)	67 (100)
*Age*			
12-18 years old	6 (85.7)	1 (14.3)	7 (100)
19-34 years old	44 (81.5)	10 (18.5)	54 (100)
35-64 years old	59 (64.8)	32 (35.5)	91 (100)
65 years old or more	2 (100)	0 (0)	2 (100)
*Underlying disease*			
Diabetic nephropathy	8 (66.7)	4 (33.3)	12 (100)
Arterial hypertension	47 (85.4)	8 (14.6)	55 (100)
Glomerulonephritis	23 (76.7)	7 (23.3)	30 (100)
Polycystic kidney diseases	6 (66.7)	3 (33.3)	9 (100)
Other	27 (56.25)	21 (43.75)	48 (100)

### Samples collection and yeasts identification

Samples containing 2 mL of saliva were collected from all patients, by previous stimulation with chewing gums. When lesions were present, a sterile swab was rubbed on the mucosal surface of the buccal cavity. Subsequently, 100 μL of cells suspensions were inoculated on the surface of Sabouraud Dextrose Agar (SDA; Oxoid, UK) added 300 μg/mL of cloramphenicol (Park-Davis), by using a Drigalsky loop. The plates were incubated at 37°C for 48 h. Yeast colonies were plated on CHROMagar Candida® (CHROMagar Microbiology, Paris, France) to check for purity and screening for different color colonies. Species identification was based on the characteristics of the cells observed microscopically after cultivation on cornmeal agar added Tween 80, as well as assimilation and fermentation testing and ID32C System (bioMérieux Marcy l’Etoile, France), whenever it was necessary [15]. Strains belonging to the *Candida parapsilosis* species complex were identified previously with molecular methods [13].

### *C. albicans* DNA extraction

*C. albicans* cells were grown overnight in YPD liquid medium (Dextrose 20 g/L, peptone 20 g/L, Yeast Extract 10 g/L) incubated at 30°C rotated at 200 rpm in a gyratory shaker (TE-420, Tecnal ® Piracicaba, Brazil). DNA was extracted using the PrepMan Ultra sample preparation reagent (Applied Biosystems, Foster City, CA) according to the manufacturer's instructions. Genomic DNA concentration and purity were checked with a NanoDrop instrument (Thermo Scientific; Amersham Pharmacia Biotech, Wilmington, DE, USA).

### Microsatellite typing PCR and ABC genotyping

Microsatellite typing was performed using the primer M13 (5’ GAGGGTGGCGGTTCT--3’) (IDT) as previously described [16]. ABC genotyping was performed with the following primers: CAINT- L (5′-ATAAGGGAAGTCGGCAAAATAGATCCGTAA-3′) and CA-INT-R (5′-CCTTGGCTGTGGTTTCGCTAGATAGTAGAT-3′) [17]. Briefly, 1.0 μL of DNA 40 ng/μL was added to 29 PCR Master Mix (Promega) to a final volume of 25 μL. The samples were amplified in a Thermocycler (Amplitherm TX96, USA) using the following cycling parameters: one initial cycle of 94°C for 3 min followed by 30 cycles of 1 min at 94°C, 1 min at 57°C, 1 min at 72°C and a final cycle of 5 min at 72°C. For Microsatellite typing, 1.0 μL of DNA 40 ng/μL, 2.5 μL of 10 × PCR buffer (100 mM Tris–HCl, pH 8.3, 500 mM KCl, 3.5 mM MgCl_2_), 5 μL of dNTPmix (100 mM each dNTP), 1.0 μL of each primer (50 pmol/μL), 0.13 μL of tween 20 and 1.0 unit of Taq DNA polymerase were added to a final volume of 25 μL. Forty-five cycles of amplification were performed using the same cycling parameters described previously, except that the annealing temperature was 36°C for the Microsatellite typing. PCR products were size-separated by agarose gel electrophoresis, and the gel was stained in a 0.5 μg/mL ethidium bromide buffer solution (TAE).

### Computer-assisted Microsatellite data analysis

Gel images were analyzed with the GelCompar II software, version 4.5 and BioNumerics (Applied Maths, Kortrijk, Belgium). The similarities between the profiles were calculated using the Dice coefficient to generate the matrixes of similarity coefficients to dendrogram constructions. For profile clustering, the unweighted pair-group method with arithmetic averages was used with a tolerance of 2%.

### Tests for hyphae formation

An initial amount of 10^6^ cells/mL in YPD + 20% FBS (Fetal Bovine Serum, Sigma-Aldrich®, Brazil) was incubated at 37°C, with gyratory shaking at 200 rpm. After 3 h incubation, samples of the cultures were mixed with an equal volume of 10% formaldehyde to arrest further development and the mean morphology index (MI) was determined [18]. Values close to 1 indicate a population of spheroidal yeast cells and values close to 4 indicate a population of true hyphal cells, with values between 1 and 4 indicating mixed or pseudohyphal morphologies.

### Phospholipase assay

Phospholipase activity was estimated by the egg yolk agar method [19], with inocula prepared from overnight NGY (Neopeptone (Difco, Detroit, MI) 1 g/L, glucose 4 g/L and yeast extract 1 g/L) cultures standardized to 2 × 10^5^ cells/mL.

### *Candida albicans* killing by polymorphonuclear neutrophils

PMN freshly isolated from blood samples of a single healthy volunteer on the day of the experiment [20] were suspended in Eagle’s minimal essential medium (Gibco) + 20 mM HEPES, pH 7.2, and standardized to 8 × 10^5^ PMN/mL. *C. albicans* cells grown overnight in NGY washed and resuspended at 5 × 10^6^ yeasts/mL in HEPES-buffered Eagle’s minimal essential medium containing one-tenth volume of fresh human plasma. Equal volumes of PMN and yeast suspensions were mixed and incubated at 37°C for 3 h with rotation at 50 rpm. The phagocytosis of *C. albicans* were established by counting in triplicate the number of yeasts inside or attached in 100 PMNs [21].

### Statistical analysis

Data were analyzed using the statistical software “GraphPad”, version 3.0. Results were presented as mean ± standard deviation, and differences were analyzed by the Mann–Whitney test. For all the analyses, P was considered a default value of 0.05 and the confidence interval of 95%.

## Results

### Patients demographic data

Patient’s clinical and demographic data are summarized in Table 1. For both regions, the majority of the patients had arterial hypertension as the most frequent underlying disease. In addition, the most frequent age class was 35 to 64 years old (Table 1).

### Microbiology profiling of *Candida* spp. in oral candidiasis

From the strains obtained from Natal, RN (Northeast), it was possible to isolate yeasts from 70 out of 111 patients (63.1%), while from Maringa, 12 out of 43 (27.5%) *Candida* spp positive cultures were obtained. Species distribution for both regions is described in Table 2. *C. albicans* was the most predominant species found in both cities, followed by *C. tropicalis*. In only a single patient from Maringa it was possible to isolate three strains belonging to different *Candida* species (*C. albicans*, *C. glabrata*, *C. tropicalis*).

**Table 2:** **Species distribution of *Candida *** **spp. isolated of oral cavity of kidney transplant recipients from two different geographic regions of Brazil: Natal, RN and Maringa, PR**

**Species**	**Natal (n,%)**	**Maringá (n,%)**	**Total (n,%)**
*Candida albicans*	59 (77.6)	17 (22.4)	76 (100)
*Candida dubliniensis*	2 (100)	0 (0)	2 (100)
*Candida glabrata*	2 (66.7)	1 (33.3)	3 (100)
*Candida tropicalis*	3 (75)	1 (25)	4 (100)
*Candida orthopsilosis*	2 (100)	0 (0)	2 (100)
*Candida metapsilosis*	2 (100)	0 (0)	2 (100)
Total	69 (78.4)	19 (21.6)	88 (100)

Of note, only one sample from each patient was collected, except for three different patients from the South region of Brazil. From patient 37, strain 06S was obtained from saliva, while strain 06 L from oral lesion. Patient 38 had two *C. albicans* strains collected from the same lesion, showing two clear different phenotypes (10S, smooth and 10R, with rough colonies). Patient 40 showed two different lesions and two strains were obtained, one from the tongue and another one from the gums (15 T and 15G, respectively) in addition to a strain obtained from saliva (15S).

### ABC typing of *Candida albicans*

DNA from all the 76 strains of *C. albicans* and the reference strains ATCC90028 and SC5314 was submitted to ABC typing. Genotype A was the most prevalent with 58 isolates (76.4%) followed by Genotype C, with 15 strains (19.27%) and Genotype B, with 3 isolates (3.9%; Table 3). The same trend was observed when the strains obtained from each region were analyzed separately: In the Northeast of Brazil, *C. albicans* genotype A was the most prevalent, with 42 isolates (71.2%), followed by Genotype C (n = 14 isolates; 23.7%) and Genotype B, with 3 strains (5.1%). The higher incidence of genotype A was also found in the South of the country with 16 isolates (94.1%) followed by genotype C (n = 1 isolate; 5.9%). *C. albicans* genotype B was not observed for the isolates obtained from this region (Table 3).

**Table 3:** **Patients enrolled in this study, geographic region ABC genotyping and virulence attributes of *Candida albicans***

**Isolate number**	**Clinical condition**	**Procedence**	**ABC typing**	**Phospholipase activity PZ (cm)**	**Morphology Index (MI)**	**No of *C. albicans *** cells phagocytosed by 100 PMNs* (%)
1	Colonization	Natal	B	0.6 ± 0.07	2.46 ± 0.70	134 ± 15.3
2	Colonization	Natal	A	0.55 ± 0.04	3.43 ± 0.77	115 ± 14.6
3	Colonization	Natal	A	0.48 ± 0.06	3.22 ± 0.64	66 ± 7.9
5	Infection	Natal	C	0.58 ± 0.02	2.23 ± 0.57	98 ± 6.7
6	Colonization	Natal	A	0.53 ± 0.01	2.24 ± 0.51	100 ± 12.5
8	Colonization	Natal	A	0.57 ± 0.04	1.86 ± 0.43	130 ± 19
10	Infection	Natal	B	0.56 ± 0.12	2.17 ± 0.62	166 ± 8.4
11	Colonization	Natal	C	0.58 ± 0.08	2.15 ± 0.77	28 ± 3.5
12	Infection	Natal	A	0.54 ± 0.02	2.46 ± 0.56	150 ± 11.1
13	Colonization	Natal	C	0.6 ± 0.03	2.17 ± 0.65	126 ± 31.6
17	Colonization	Natal	A	0.52 ± 0.03	3.63 ± 0.63	98 ± 20.2
20	Colonization	Natal	A	0.44 ± 0.01	2.85 ± 0.81	86 ± 21.2
21	Colonization	Natal	A	0.4 ± 0.06	2.78 ± 0.92	114 ± 21.5
23	Colonization	Natal	C	0.53 ± 0.09	2.45 ± 0.67	101 ± 11.4
24	Infection	Natal	A	0.51 ± 0.10	3.05 ± 0.74	132 ± 13.9
28	Infection	Natal	A	0.55 ± 0.04	2.84 ± 0.80	165 ± 9.5
30	Colonization	Natal	C	0.52 ± 0.04	2.17 ± 0.62	119 ± 20
31	Colonization	Natal	A	0.49 ± 0.03	2.64 ± 0.82	129 ± 15
32	Colonization	Natal	A	0.49 ± 0.12	2.81 ± 0.65	123 ± 13.2
34	Colonization	Natal	A	0.52 ± 0.07	2.36 ± 0.64	156 ± 16.5
37	Colonization	Natal	B	0.57 ± 0.01	3.38 ± 0.80	124 ± 19.1
40	Infection	Natal	A	0.51 ± 0.06	3.7 ± 0.56	142 ± 15.9
41	Colonization	Natal	A	0.54 ± 0.03	2.7 ± 0.69	172 ± 21.1
44	Infection	Natal	A	0.48 ± 0.03	2.8 ± 0.77	176 ± 11.6
46	Colonization	Natal	A	0.46 ± 0.04	2.81 ± 0.77	172 ± 20.1
50	Colonization	Natal	A	0.54 ± 0.04	3.24 ± 0.88	157 ± 36.6
51	Colonization	Natal	A	0.51 ± 0.02	2 ± 0.43	130 ± 19.5
53	Colonization	Natal	C	0.53 ± 0.09	2.79 ± 0.71	132 ± 15.9
54	Colonization	Natal	A	0.47 ± 0.02	1.8 ± 0.40	132 ± 9.2
60	Colonization	Natal	C	0.57 ± 0.03	2.15 ± 0.36	146 ± 9
61	Colonization	Natal	A	0.49 ± 0.01	2.48 ± 0.75	142 ± 9.2
70	Colonization	Natal	C	0.51 ± 0.03	2.99 ± 0.50	140 ± 12.7
72	Colonization	Natal	C	0.46 ± 0.04	2.31 ± 0.61	136 ± 9
82	Colonization	Natal	C	0.55 ± 0.03	3.36 ± 0.93	130 ± 13.2
85	Colonization	Natal	C	0.56 ± 0.04	3.15 ± 0.83	144 ± 9.5
107	Colonization	Natal	C	0.57 ± 0.01	2.39 ± 0.63	156 ± 10.1
06A	Colonization	Maringá	A	0.5 ± 0.02	2.34 ± 0.71	80 ± 17.4
06 L	Infection	Maringá	A	0.56 ± 0.07	2.94 ± 0.58	75 ± 8.7
10Li	Colonization	Maringá	A	0.55 ± 0.01	2.42 ± 0.70	176 ± 6.1
10S	Colonization	Maringá	A	0.64 ± 0.01	2.54 ± 0.76	105 ± 14.7
12A	Colonization	Maringá	A	0.49 ± 0.02	3.3 ± 0.79	59 ± 16.8
12 L	Infection	Maringá	A	0.51 ± 0.05	3.2 ± 0.68	90 ± 13.2
15A	Colonization	Maringá	A	0.42 ± 0.02	2.96 ± 0.83	116 ± 27.1
15G	Infection	Maringá	A	0.42 ± 0.03	3.13 ± 0.81	86 ± 21.9
15 L	Infection	Maringá	A	0.47 ± 0.08	2.58 ± 0.68	76 ± 16.5
18A	Colonization	Maringá	C	0.56 ± 0.11	3.05 ± 0.67	102 ± 11.2
111 L	Colonization	Natal	C	0.55 ± 0.03	2.6 ± 0.70	80 ± 17.4
111R	Colonization	Natal	C	0.6 ± 0.15	4 ± 0.00	11 ± 3.2
ATCC90028		Reference	A	0.7 ± 0.08	3.82 ± 0.5	158 ± 17.5
SC5314		Reference	A	0.52 ± 0.04	3.28 ± 0.81	137 ± 20.6

The isolates were obtained from the oral cavity of kidney transplant recipients from Natal, RN and Maringa, PR, Brazil.

### *Candida albicans* microsatellite typing versus ABC genotyping

**Figure 1:**
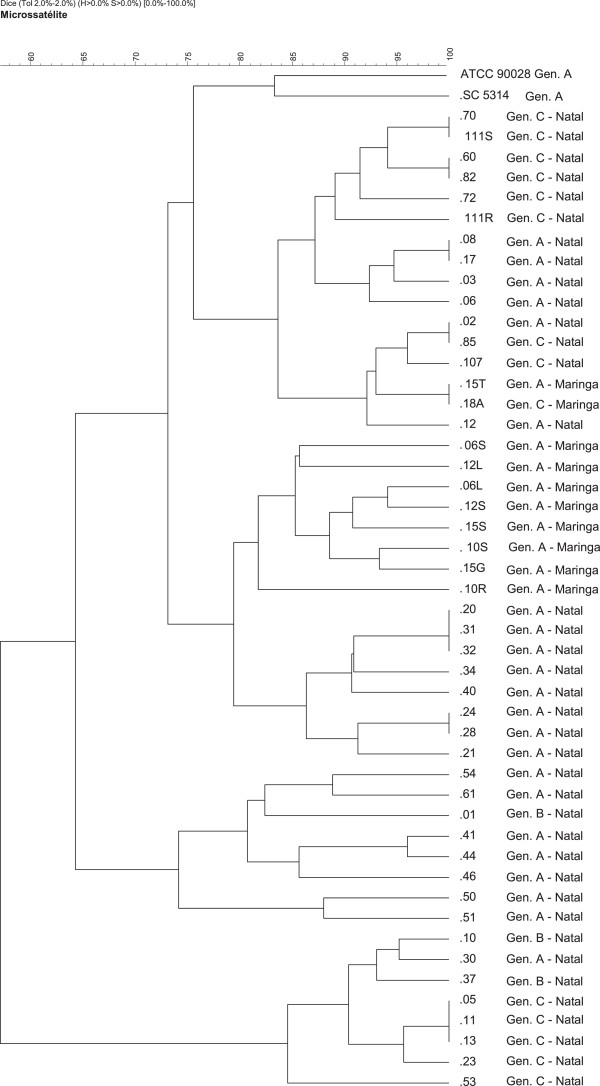
**Unweighted pair-group method with arithmetic averages dendrogram with 2% of tolerance of 50 strains of *Candida albicans *** **clinical isolates.**

We randomly selected 48 isolates of *C. albicans* including some strains belonging to genotype A (21 from Natal and 10 from Maringa) an all the other strains belonging to genotypes B and C from both regions and performed Microsatellite genotyping. The genomic DNA of all of the 48 isolates was successfully amplified with the M13 primer, which targets genome repetitive sequences. The DNA amplification generated well-defined band patterns, ranging from 100 pb to 2 kb. The microsatellite technique showed sufficient discriminatory power for recognizing intraspecific variation (Figure 1). As expected, the external control strains of *C. albicans*, SC5314 and ATCC90028, were placed in completely different clusters by the dendrogram analysis performed using GelCompar II, proving the efficiency of the technique in separating strains isolated from different geographic areas. These control strains showed 75% similarity and were placed together within a different cluster of all the other clinical isolates (Figure 1).

We could not detect any particular cluster of either colonizing or infecting strains. In addition, in both cases, strains with 100% similarity obtained from different patients were found. Regarding to the comparisons of genetic relatedness within the two different geographic regions of Brazil, we could observe an enriched cluster for Maringa (South), where 80% of the strains were placed together. In addition, they ranged from 80% up to 93% similarity among them.

Other interesting finding is associated with some degree of correlation observed between the ABC type of *C. albicans* and the high percentage of similarity among these isolates when Microsatellite primer M13 was used. In most of the cases, strains with identical percentage of similarity determined with primer M13, also had the same A or C genotype (except for strains 02 and 85 from Natal as well as 15 T and 18A from Maringa; A and C genotypes in both cases). In addition, *C. albicans* genotype C strains from Natal were placed within two well-defined separated clusters with proven high relatedness (more than 90% similarity within the same cluster). In relation to genotype B, two out of three isolates showed a similarity of 94%, being placed in the same cluster (Figure 1).

Furthermore, when different strains were obtained from different samples of the same patient, they all had the same ABC genotype (genotype A). Nevertheless, Microsatellite using primer M13 showed higher discriminatory power, because these strains were not considered identical with the latest technique, showing some degree of genetic variability, when this method was used.

### Virulence attributes of *Candida albicans* A, B and C genotypes

For all the virulence factors tested, we only performed statistical analysis for comparisons among strains belonging to genotypes A and C, because of the low number of isolates belonging to genotype B found. In relation to phospholipase production, all the strains were able to produce the enzyme, regardless if they were obtained from episodes of colonization or infection. For genotype A, a Mean Pz of 0.51 ± 0.04 was obtained, while for genotype C, a Mean of 0.55 ± 0.05, while for the genotype B, the Mean Pz was 0.57 ± 0.07. No statistical differences were found when strains belonging to genotypes A and C were compared (Table 3).

When the morphology index (MI) was used to score cells morphology, no clear differences could be observed for the three different A, B or C genotypes. The ability of the strains to change the morphology from bud cells to hyphae when grown in the presence of YPD + 20% FBS was verified for strains belonging to the three referred genotypes. Cells were predominantly found as pseudohyphae, with a mean MI ranging from 2.66 ± 0.61 (genotype C) to 2.77 ± 0.69 (genotype A). The mean MI isolates of genotype B was 2.67 ± 0.71. No statistical differences were observed among the isolates (Table 3).

We also investigated the ability of the different strains to resist to phagocytosis by PMNs. Therefore, the number of *C. albicans* cells phagocytosed by 100 PMNs was determined after three hours of co-incubation of both cells. *C. albicans* genotype C was more resistant to PMNs attack. Strains belonging to genotype B had an average of 141.33 ± 14.27 cells phagocytosed by 100 PMNs. Strains belonging to genotype C were significantly more resistant to the attack of phagocytic cells than the isolates belonging to genotype A (Mean of 109.93 ± 12.29 versus 121.67 ± 16.06, respectively; Table 3).

Virulence attributes of *Candida albicans* strains obtained from two different geographic regions of Brazil (Northeast and South).

When the pathogenicity factors were compared among all the strains obtained from the two geographic regions, the strains obtained from Maringa (South) expressed more efficiently all the virulence factors evaluated in vitro. This difference was considered statistically significant for resistance to phagocytosis by PMN (Table 4). Interestingly, 40% of the isolates obtained from Maringa were obtained from lesions, whereas only 18.4% of the isolates from Natal were infecting (Tables 3 and 4).

**Table 4:** **Virulence attributes of *Candida albicans *** **isolates obtained from the oral cavity of kidney transplant recipients of two geographic regions of Brazil: Natal, RN and Maringa, PR**

	**Natal vs Maringa**
**Phospholipase activity PZ (cm)**	0.53 ± 0.05 vs 0.51 ± 0.04
**Morphology index MI**	2.70 ± 0.65 vs 2.85 ± 0.72
**No of *C. albicans *** cells phagocytosed by 100 PMNs**	125.61 ± 14.61 vs 96.50 ± 16.36*

*Statistically significant; P < 0.05. **PMNs- = polymorphonuclear neutrophils.

## Discussion

In our study including 154 patients with oral candidiasis, the vast majority of isolates were *C. albicans*. This is in accordance with other studies that although report an increase of Non-*Candida albicans Candida* species, *C. albicans* is still predominant [13,22-24].

Because *C. albicans* is the most virulent and frequently isolated species of the genus *Candida* from oral candidiasis, we phenotypically characterized three attributes of virulence (extracellular activity of phospholipase, morphogenesis and resistance to phagocytosis by PMNs) of 48 clinical isolates of this species obtained from the oral cavity of 154 kidney transplanted patients from two different regions of Brazil (Northeast and South). In addition, we investigated genetic relatedness of these strains with two different methodologies of genotyping (ABC typing and Microsatelitte technique with M13 primer).

We found in the present study a high incidence of *C. albicans* isolates belonging to genotype C from the oral cavity of kidney transplant recipients. Nevertheless, *C. albicans* genotype A was still the most prevalent. The majority of other series upon oral candidiasis or oral colonization investigating *C. albicans* ABC genotypes with the same method have found genotype A as the most prevalent type [9,24,25]. *C. albicans* Genotype A is also predominant in other series with strains obtained from different body sites, including cases of systemic infection [17,26-29].

Interestingly, we found a high incidence of strains belonging to genotype C in our study*.* This seems to be an unusual finding. For instance, in the study of Sardi et al [8], genotype C was not found. The same findings were observed by Jacobsen et al. [30]. In a total of 32 individuals with *C. albicans* oral colonization, oral candidiasis and other superficial infections, only one strain belonging to genotype C was found. Actually, Odds et al. [28] described genotype C as a rare genotype, found in only 173 isolates of 1931 *C. albicans* obtained from different sources of the world. To the best of our knowledge, this is the largest population study on *C. albicans* typing.

Another intriguing finding is that, except for two cases, all the type C strains were placed together within two well-defined separated clusters in the dendrogram. Mccullough et al [17] hypothesized that *C. albicans* genotype C is probably genotype A that probably acquired an intron. This may partially explain why they were considered identical by Microsatellite technique in two occasions. Taking together, these data suggest the peculiarity of the distribution of genotypes of *C. albicans* isolates from the oral cavity of kidney transplant patients, firstly analyzed in our study.

Microsatellite genotyping using primer M13 showed satisfactory discriminatory power and may be applied for epidemiological studies of *C. albicans* infection with low cost and good effectiveness. This technique has not been widely used for this species [21], but has been largely used to type *Cryptococcus neoformans*[16,31].

The fact that we have found some strains obtained from different patients considered undistinguishable (100% similarity) is not unusual. Other authors have described that it is possible that unrelated strains share the same genotype. For instance, Sampaio et al [32] using Microsatellite technique with CAI4 locus found that in 73 independent vaginal isolates, 44 genotypes could be detected. In addition, we could not completely rule out the possibility of strain cross-contamination while their staying in Hospital.

One of our remarkable findings was the placement of most of the strains from the South of Brazil within the same cluster. They also shared the same ABC type (genotype A). This fact may be partially explained due to *C. albicans* co-evolution with humans in determined geographic regions once this species belongs to the normal oral microbiota [28].

We found some degree of correspondence of strains genetic relatedness when both genotyping methodologies were applied. Only in two occasions, strains with different A or C types were designated as identical with Microsatellite technique. This difference reinforces the necessity of a combination of at least two different techniques for *C. albicans* strains typing [33].

Besides the fact we have found a high rate of type C strains of *C. albicans*, these strains were also statistically more resistant to phagocytosis by PMNs than genotypes A and B strains. Although at the moment of strains collection only one genotype C strain was obtained from patients with lesions, the fact that they were immunosuppressed individuals and colonized by possibly virulent strains that were able to highly express the virulence factors evaluated in vitro may influence future clinical outcomes.

Related to the virulence factors expressed by the isolates from Natal and Maringa, it was possible to observe that the isolates from the South of Brazil were markedly resistant to phagocytosis by PMNs. A recent study regarding the characterization of oral biofilms formed on mucosal surfaces found that a strong migration of PMNs which formed intense nests of aggregates juxtaposed to mucosal biofilms. In addition, in sites with thicker biofilms, neutrophils migrated through the entire width of the mucosa, reinforcing the importance of specific white cells also in oral candidiasis [34].

## Conclusions

A limitation of our study was the low number of isolates, specifically belonging to genotype B. Nevertheless we could demonstrate the peculiarities of *C. albicans* strains obtained from the oral cavity of kidney transplant recipients of two different regions of Brazil. Considering that this country has continental dimensions, we found that most of the strains of each different region were placed in the same cluster and were markedly resistant to phagocytosis by PMNs. In addition, we also found a high rate of genotype C (a rare genotype) strains isolated from the oral cavity of this group of patients. To the best of our knowledge, this study is the first one to characterize oral *C. albicans* strains of kidney transplant recipients from two different regions of a continental size country (Brazil).

## Abbreviations

C: *Candida*; spp: Species; PMN: Polymorphonuclear; YPD: Yeast peptone dextrose; g/L: Grams per liter; °C: Celsius degrees; ATCC: American Type Culture Collection; SDA: Sabouraud dextrose agar; UK: United Kingdom; μg/mL: Micrograms per milliliter; h: Hour; DNA: Deoxyribonucleic acid; rpm: Revolutions per minute; CA: California; DE: Delaware; USA: United States of America; PCR: Polymerase chain reaction; G: Guanine; A: Adenine; C: Cytosine; T: Thymine; IDT: Integrated DNA technologies; μL: Microliter; ng/μL: Nanograms per microliter; mM: Millimolar; HCl: Chloridric acid; pH: Potencial hydrogen; KCl: Potassium chloride; MgCl2: Magnesium chloride; dNTP: pmol/μL, picomoles per microliter; FCS: Fetal calf serum; MI: Morphology index; NGY: Cells/ml. cells per microliter; HEPES: PMN/mL, polymorphonuclear per microliter; bp: Base pairs; Pz: Phospholipase zone.

## Competing interests

The authors declare that they have no competing interests.

## Authors’ contributions

WP and GM analyzed data, performed research; wrote the paper. VL permormed research, TI and EP, collected samples. All authors read and approve the final manuscript.

## Authors’ information

GM, Professor of Clinical Microbiology, Dept. de Análises Clínicas e Toxicológicas, Universidade Federal do Rio Grande do Norte, Brazil and Leader of the Laboratório de Micologia Médica e Molecular.

## Pre-publication history

The pre-publication history for this paper can be accessed here:

http://www.biomedcentral.com/1472-6831/14/20/prepub
